# Day case laparoscopic cholecystectomy: a review of patient selection factors and identification of potential barriers to same‐day discharge

**DOI:** 10.1111/ans.19241

**Published:** 2024-10-09

**Authors:** Jamie Rickward, Iman Hameed, Simon Ho, Shiran Wijeratne

**Affiliations:** ^1^ University of Notre Dame Sydney Sydney New South Wales Australia; ^2^ Werribee Mercy Hospital Melbourne Victoria Australia

**Keywords:** cholecystectomy, day, laparoscopic, patient discharge, patient satisfaction, patient selection, surgery

## Abstract

**Background:**

Day‐case laparoscopic cholecystectomy (DCLC) is a useful tool for minimizing hospital admissions and prolonged presurgical wait times in suitable patient cohorts. There have been many international studies to support this finding and an increasing interest has grown in implementation in Australia. This review aims to provide clarity how to best implement this tool in gallbladder disease patient demographic.

**Observations:**

This literature review evaluates studies on day‐case cholecystectomy procedures, focusing on patient factors, procedural aspects, surgical morbidity, and systemic implications. It explores inclusion and exclusion criteria for day‐case suitability, factors influencing same‐day discharge, reasons for hospital admission, pain management, patient quality of life, patient satisfaction, and cost implications.

**Conclusions:**

DCLC, when selected judiciously, is a safe alternative to overnight stay procedures for cholecystectomy with comparable surgical outcomes and patient satisfaction, affirming its viability. Strict patient selection criteria can aid in optimizing the successful implementation procedure, reducing unexpected admissions and readmissions and we have demonstrated useful criteria for guidance in establishing day‐case laparoscopic cholecystectomy protocol at a hospital.

## Graphical abstract

This review synthesizes international studies on day‐case laparoscopic cholecystectomy (DCLC), highlighting patient selection criteria, procedural factors, and outcomes. Findings indicate that judicious selection of patients for DCLC can offer a safe alternative to overnight stays, with comparable surgical outcomes and high patient satisfaction. Strict adherence to selection criteria can optimize implementation, reducing unexpected admissions and readmissions, thus providing valuable guidance for establishing DCLC protocols in hospitals.

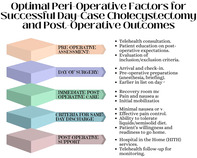

## Introduction

Gallstone disease is a common presentation with significant demand on the hospital system to perform cholecystectomy procedures in a safe, efficient, and cost‐effective manner.^1^

In Australia during 2020–2021 there were 60 393 cholecystectomies performed, with 57 591 or 95% of these requiring overnight admission.^2^ Of these 50% were performed electively with an average time to surgery from waiting list booking of 56 days.^3^ This is further compounded by most patients requiring an overnight admission.^4^ In Australia for 2021–2022 there were 2 459 622 surgical procedures performed.^5^ Of these procedures, 47 816 were discharged on the same‐day.^5^ There is a substantial opportunity for optimization and, through appropriate patient selection, a transition to a greater focus on same‐day procedures.

The primary issue of concern is whether day‐case laparoscopic cholecystectomy (DCLC) is safe for patients with concerns of delayed detection of severe adverse events. Additionally, there are apprehensions regarding the effectiveness of post‐operative pain management in a home setting.^6^

Multiple meta‐analyses have suggested that for most patients, DCLC is safe and suitable.^6^, ^7^ Appropriate preoperative patient selection for DCLC can help to alleviate unnecessary hospital admissions and reduce surgical wait times.^8^ The scope of this review is to better understand which criteria are most appropriate in successfully implementing a DCLC protocol and the factors which may predict the need for an overnight admission and unplanned overnight admission.

## Methods

English‐language reports related to day‐case cholecystectomy procedures were sought using the PubMed and Cochrane databases. The search process was conducted by two independent investigators to ensure a comprehensive and unbiased selection of studies. Studies were included if they compared day‐case and overnight cholecystectomy to understand the clinical decision‐making process. The search covered publications from 1 January 2000 to 31 December 2021, with seminal studies published before 2000 included when relevant and when more recent data was not available.

A total of 352 studies were initially identified. The selection process involved an initial abstract inspection of all identified studies, followed by a full‐text review of 112 studies that met the initial criteria based on abstract screening. Finally, 47 studies were included in the review based on specific inclusion and exclusion criteria.

The inclusion criteria were:

- Studies comparing day‐case and overnight cholecystectomy.
- Publications in English.
- Studies involving adult patients undergoing cholecystectomy.
- Peer‐reviewed articles including randomized clinical trials, meta‐analyses, systematic reviews, case‐based, and observational studies.

The exclusion criteria were:

- Non‐English publications.
- Studies focusing solely on paediatric populations.
- Articles without sufficient methodological rigour or relevance to the review's objective.

The 47 selected studies included 2 Cochrane reviews, 8 randomized clinical trials, 2 meta‐analyses, 3 systematic reviews, 3 national or international guidelines, and 29 case‐based and observational studies.

### Patient factors: day case suitability

#### Inclusion criteria for day‐case procedure

The selection standards for day‐case cholecystectomy are aimed at optimizing patient, clinician, and hospital factors to streamline the workflow.^6^, ^9^, ^10^ Over time, there have been advancements and improvements in patient selection criteria due to a greater body of research and collective experience.

Calland *et al*. showed that the success rate of day‐case laparoscopic cholecystectomies can increase from 21 to 72% with the appropriate selection of patients suitable for day surgery.^11^ Metcalfe *et al*. demonstrated a comparable success rate of 76% for same‐day discharge by adhering to meticulous selection criteria.^12^ Multiple studies have commented on factors which could contribute to a standard inclusion criterion, these common criteria have been synthesized into a standard inclusion criterion and can be found below in Table 1.

**Table 1:** Optimal inclusion criteria for day‐case cholecystectomy based on unifying literature.

Criteria	Description	References
Elective procedure	Non‐emergency surgery	6, 9, 10, 11, 12, 13, 14, 15, 16
Age below 65	Preferred age	6, 7, 9, 12, 13, 15
Absence of significant underlying medical conditions	Reduced likelihood of complication due to the absence of major comorbidities	6, 7, 11, 12, 15
ASA grade I or II	Low anaesthetic risk	7, 13, 14, 15, 16
No prior upper abdominal operations	Virgin abdomen less likely to require alternative approach due to previous operative sites.	6, 7, 9, 12, 13
“No/low” risk for common bile duct stones	Based on radiographic and biochemical findings	6, 9, 10, 11, 12, 20
Proximity to hospital	Patient lives close enough to return to the hospital quickly in the case of an emergency post‐discharge	7, 8, 13, 15
Responsible adult at home post‐discharge	Presence of a caregiver at home.	7, 8

Standard day case laparoscopic cholecystectomy inclusion criteria as per evidence base. While considered ideal, this list may not be used as an ‘absolute’ inclusion criterion. Please note that this table may not capture all studies related to each specific inclusion criterion. Also, ‘Empirical approach’ signifies that the inclusion criterion was established through general clinical practice and not attributed to a specific study.

Empirical approaches were used to establish the primary requirements for the ideal patient, which included elective procedures case, age below 65, absence of significant underlying medical conditions, no prior upper abdominal operations, and a “no/low” risk for common bile duct stones. The latter criterion was based on two preoperative studies, namely a maximum common bile duct diameter of no more than 5 mm on preoperative ultrasound and normal biochemical results.^6^, ^7^, ^9^

Voyles *et al*. noted that patients were typically chosen for day surgery based on their suitability for the procedure (i.e., normal healthy individuals or those with mild systemic disease), proximity to the hospital, and the presence of a responsible adult to care for them post‐discharge.^9^

Similar inclusion criteria were used by Hollington *et al*. and Keulemans *et al*. both adding peri‐operative anaesthetic risk to their assessment, including an ASA score as part of their criteria.^13^, ^14^ There has been a greater degree of tolerance for poorer ASA grades as the knowledge base broadens. Kumar *et al*. study, like most others' on this topic, deemed only ASA Grade I and II patients to be suitable candidates.^15^ However, Mjaland *et al*. demonstrated that some ASA Grade III patients could also undergo DCLC successfully.^16^

This sentiment was echoed by Pham *et al*. who stated inclusion/exclusion criteria became more flexible with increasing clinician experience.^8^ Specifically, ASA grade, age, and use of anticoagulants were no longer considered an absolute contraindication for DCLC. However, despite the implementation of a unit protocol, there still exists variability in patient selection and management, which ultimately reflects differences in surgeon preference and experience.^8^

Utilization of telehealth as an adjunct to day case procedures has also been reviewed and found that the implementation of preoperative telehealth patient clinics could further reduce the need for patients to present to hospital.^17^ Urbonas *et al*. suggested that most patients who were deemed as suitable for a day case, that is, a benign gallbladder pathology, were found also to be suitable for a pre‐surgical consultation without the need for a pre‐anaesthetic clinic.^17^

**Fig. 1:**
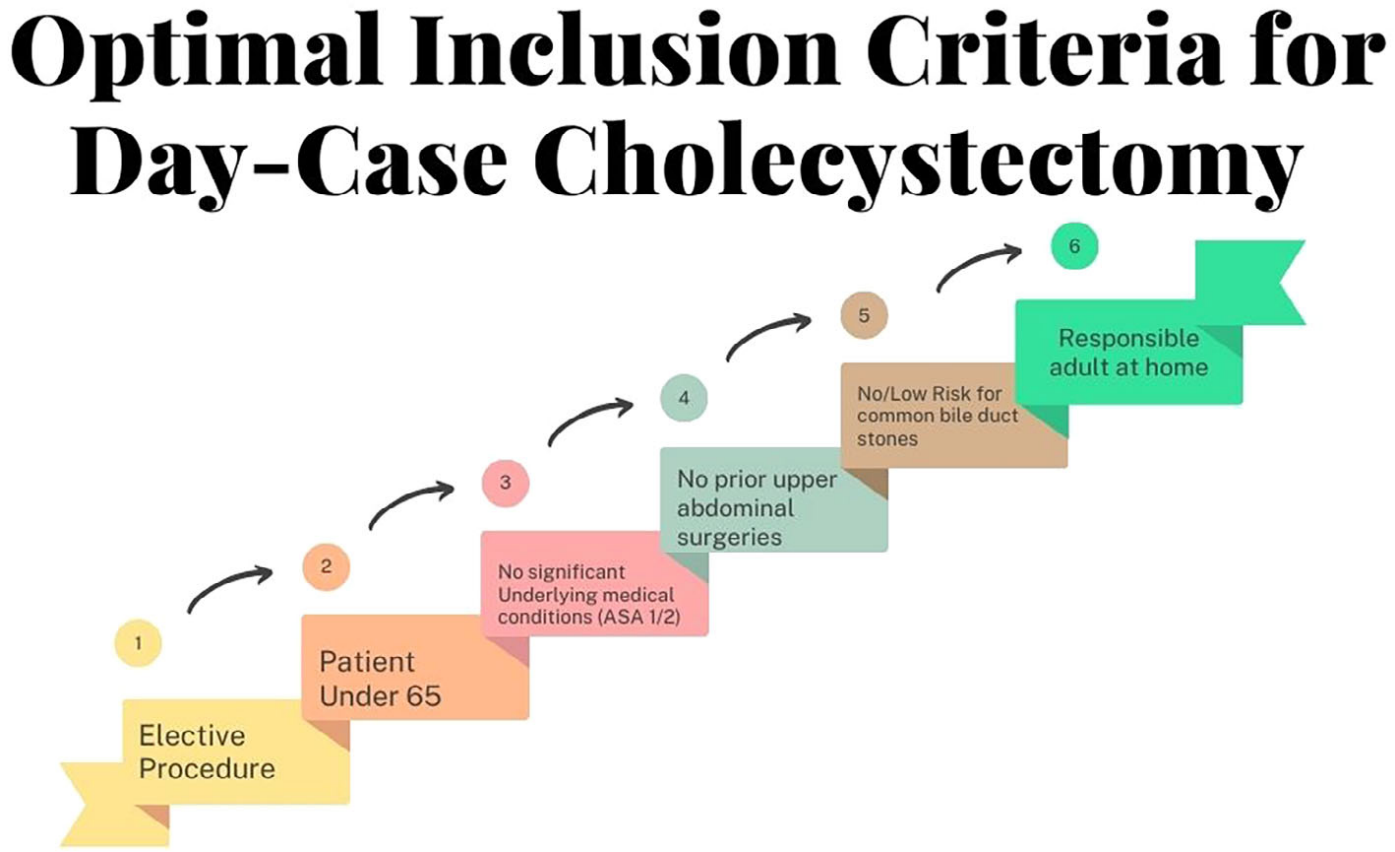
Optimal patient inclusion criteria flowchart.

Many of these factors contribute to the sentiment that tertiary facilities are better equipped to enact day case procedures.^8^ This is due not only to the availability of more resources for patient assessment, risk stratification, and surgical theatres, but also to the presences of highly trained clinicians.^18^ High‐volume abdominal general surgeons and hepatobiliary surgeons at tertiary centres often possess greater specialized skills and experience.^19^ The procedural skills are just as crucial in ensuring successful outcomes. Indicating that both the advanced, or more abundant, resources of tertiary facilities, in combination with expertise and volume of procedures both play their part in the success of day procedures.^19^ A summary of optimal inclusion criteria is demonstrated in Figure 1.

#### Exclusion criteria for day‐case procedure

Following the advent of the DCLC, Keulemans *et al*. suggested criteria that recommended excluding patients with any of the following: an ASA score of 3 or 4, age over 70, extensive previous abdominal surgery, clinical suspicion of common bile duct stone, acute cholecystitis, and patients with calcified gallbladders.^14^ A similar exclusion criterion was suggested by Hollington *et al*. with the addition of suggesting that patients who were at significant risk of requiring conversion to open procedures should be excluded.^13^

Prior reviews demonstrating safety and efficacy of DCLC commonly excluded patients if they had a history of upper abdominal surgeries, suspected common bile duct stones, or acute cholecystitis.^7^, ^20^

Kumar *et al*. excluded patients with uncontrolled systemic disease (diabetes, hypertension), an elevated risk of intraoperative complications (non‐virgin abdomen, obesity), or a need for other interventions during the procedure.^15^ Similarly, Zaafouri *et al*. excluded patients if there was a decision to convert to laparotomy, or if there were intraoperative findings of acute cholecystitis, significant bleeding or bile leak, or a need for drain insertion.^21^

Obesity is commonly considered a risk factor during anaesthesia and perioperative surgical procedures.^22^ Many day‐case facilities implement protocols that limit day‐case cholecystectomy for individuals with a high BMI.^23^ Current literature suggests that BMI has a minimal impact on the likelihood of a successful day case with multiple cross‐institutional studies observing that BMI alone does not constitute an adequate exclusion criterion.^24^, ^25^ Additionally, it was observed by Tandon, *et al*. that there was no significant variation in day case completion across BMI stratified group.^25^ Overall, the literature indicates that while obesity should often be considered a risk in the context of the patients' entire well‐being, it is suggested that BMI should not be a sole determining factor in the exclusion of patients from DCLC.^25^ Further consideration should be given to the patient as a whole and their suitability for laparoscopic surgery, including specific technical difficulties in this group of patients such as the placement of trocars, liver retraction, and anatomical dissection of Calot's triangle.^22^

#### Factors likely to improve same‐day discharge

In their Cholecystectomy As A Day‐case (CAAD) study, to determine a standardized approach to improving same‐day discharge, El‐Sharkawy, *et al*. identified factors that may increase the likelihood of same‐day discharge. They found that younger age and lower ASA scores were associated with fewer comorbidities and anaesthetic risks, requiring less intensive perioperative monitoring, resulting in a higher probability of same‐day discharge.^19^

Educating patients about potential postoperative symptoms such as pain, nausea, and vomiting can have a significant impact on the success rate of same‐day discharge, in addition to patient selection.^26^ Al‐Qahtani *et al*. found in their randomized control trial that incorporating patient education as part of preoperative patient assessment helped guide patient expectations, resulting in a higher rate of same‐day discharge.^27^

#### Factors likely to hinder same‐day discharge

Potential factors found that may prevent same‐day discharge include intraoperative complications, such as common bile duct exploration, conversion to open cholecystectomy, and additional procedures, such as adhesiolysis.^28^ Patients aged over 50 years may experience reduced same‐day discharge rates with a 67% decrease rate of same‐day discharge (30).

Certain factors that can lead to the failure of DCLC and increased admission rate may not be identifiable or preventable through preoperative assessment. Such factors include complex anatomical dissection, persistent post‐operative pain, nausea, and vomiting.^27^

### Procedural factors: day‐case cholecystectomy versus overnight stay

#### Surgeon experience

The lead surgeon's experience and expertise in performing laparoscopic cholecystectomies is proposed to have a positive association with the success of same‐day discharge.

El‐Sharkawy *et al*. proposed that surgeons specializing in upper gastrointestinal and hepatopancreatic biliary (HPB) procedures have a higher likelihood of achieving same‐day discharge, with an observed odds ratio of −0.888 (0.798–0.989) when comparing surgeons with a HPB specialty and non‐specialist surgeons.^19^

Al‐Qahtani *et al*. showed that when procedures were carried out by consultant surgeons, they were likely to be significantly shorter in duration.^27^ In addition to this point, they also demonstrated that there is a significantly fewer number of cases performed by consultants, with the vast majority performed by high‐level surgical trainees.^27^

Overall, it seems to follow a logical sense that specialist surgeons have a higher level of proficiency than non‐specialist.^29^ This underscores the importance of surgeon expertise and institutional support in facilitating successful same‐day discharge following laparoscopic cholecystectomy. While surgical experience is likely to have some effect in reducing operative time, DCLC remains safe even when conducted by non‐specialist and trainee surgeons, provided adequate support and protocols are in place.

#### Post‐operative evaluation and same‐day discharge criteria

Post‐operative evaluation helps to ensure discharge readiness. Xiong *et al*. meta‐analysis suggested the most common post‐operative complications were pain, nausea and vomiting, diarrhoea, wound infection and associated fever^20^ but should not be considered barriers to a same‐day discharge as they can be safely managed at home.

Specialized day case pathways such as hospital in the home (HITH) services, and post‐operative telehealth consultations can help to reduce the circumstances where patients would require an admission to hospital. Many facilities now employ a dedicated day‐case pathway to ensure successful discharge which includes strict evaluation of patient's immediate post‐op complications such as pain and nausea in recovery bay, along with same‐day evaluation at home either in‐person on via telehealth. This has been shown to be a successful strategy in reducing patient re‐admission rates,^16^, ^30^, ^31^ with Akoh *et al*. demonstrating a readmission rate as low as 5.2% for DCLC.^32^

Scheduling procedures as earlier in the list/day may also accommodate a greater rate of same‐day discharge by providing adequate time post‐operatively to adequately assess if patient is suitable for discharge.^32^

Potential post‐operative evaluation and discharge criteria should consider; minimal nausea or vomiting, pain control or minimal pain, the ability of patients to tolerate a liquid or semisolid diet orally, no significant toileting difficulties and the patient's willingness to go home.^28^, ^30^, ^31^, ^33^

#### Surgery related morbidity

Surgical morbidity associated with laparoscopic cholecystectomy can be broadly categorized into major surgical morbidity and minor immediate post‐operative complaints.

##### Major surgical morbidity

Major surgical morbidity includes complications that are more serious and may require significant medical intervention. These can encompass bile duct injury, wound infection, re‐operation, intra‐abdominal collection, haematoma, postoperative pancreatitis, retained common bile duct stones, and conversion to open cholecystectomy.^11^, ^15^ Sandblom *et al*. have shown that the occurrence of these major complications is relatively rare.^34^

- Bile Duct Injury: This is a serious complication that can have significant long‐term consequences. However, the incidence rate is low and comparable between day‐case and inpatient procedures.
- Wound Infection and Re‐operation: These complications occur infrequently and are manageable with appropriate surgical and postoperative care.
- Intra‐abdominal Collection and Hematoma: These are rare and can typically be managed without the need for prolonged hospitalization.
- Postoperative Pancreatitis and Retained Common Bile Duct Stones: These complications are also infrequent and do not show increased incidence in day‐case procedures compared to inpatient surgeries.
- Conversion to Open Cholecystectomy: While conversion is sometimes necessary, it does not significantly differ in frequency between planned day‐case and inpatient procedures.

The Cochrane review by Gurusamy, *et al*. found no statistically significant difference in overall morbidity (RR 1.26, 95% CI 0.54 to 2.94) or morbidity occurring after discharge (RR 1.23, 95% CI 0.44 to 3.46) between day‐case and inpatient cholecystectomy procedures.^7^

#### Minor immediate post‐operative complaints

Minor immediate post‐operative complaints are more common and can include symptoms such as pain, nausea, vomiting, and diarrhoea. While these issues can impact the immediate post‐operative period, they are generally manageable and do not preclude same‐day discharge in most cases.^6^

- Pain: Effective pain management protocols are essential to address post‐operative pain, which is the most common reason for delayed discharge. Local anaesthetic techniques, such as the use of ropivacaine, have been shown to improve pain management and facilitate same‐day discharge.
- Nausea and Vomiting: Preoperative and intraoperative antiemetic protocols can significantly reduce the incidence of nausea and vomiting, making it possible for patients to be discharged the same‐day.
- Diarrhoea and Wound Infection: These can typically be managed with appropriate outpatient care and follow‐up.

Vaughn *et al*. suggest that these minor complaints can be safely managed at home with the support of telehealth and home‐based care services, thereby reducing the need for overnight hospital stays.^6^

With proper patient selection, effective management of minor post‐operative symptoms, and institutional support, the rates of major surgical morbidity remain low and comparable to inpatient procedures.^20^ This reassures that DCLC is a viable and safe option for appropriately selected patients.

#### Reasons for hospital admission and readmission after DCLC

The decision to admit or readmit patients following DCLC is influenced by several factors. This section outlines the primary reasons for unexpected hospital admissions and readmissions, based on current evidence and clinical practice.

Initial Hospital Admissions:

The Cochrane review by Gurusamy, *et al*. indicates that the proportion of patients requiring an unexpected extension of hospital stay was similar in both the day‐case and overnight stay groups, at 19.5% and 20.1%, respectively.^7^ The primary reasons they found for prolonged hospitalization include:

- Pain: Postoperative pain is the most common reason for extended hospital stays. Effective pain management protocols are crucial to minimize this issue.
- Nausea and Vomiting: These symptoms often result from anaesthesia and can delay discharge if not adequately controlled.
- Conversion to Open Cholecystectomy: This surgical decision is sometimes necessary due to complications or intraoperative findings, requiring longer hospitalization.
- Clerical Errors: Administrative issues can also inadvertently extend a patient's hospital stay.

Prolonged Hospital Stays:

The duration of hospital stays for patients requiring extended admission was comparable between day‐case and overnight stay groups, indicating that the severity of conditions necessitating prolonged hospitalization does not differ significantly between the two.^7^

Unplanned Admissions:

Routine DCLC performed without specific patient selection has previously resulted in higher unplanned admission rates as demonstrated by Akoh *et al*.^32^ Adequate presurgical screening using inclusion and exclusion criteria can optimize the procedure and significantly reduce unexpected admissions.^7^ Strict selection and exclusion criteria have been shown to reduce unplanned admissions and readmission rates.^35^

Readmissions:

Unplanned readmissions after DCLC are relatively uncommon but can occur.^27^ Key reasons for readmission include:

- Significant Pain: Persistent or severe pain is a leading cause of readmission. Effective pain management and patient education on pain control at home are critical.
- Nausea and Vomiting: Uncontrolled postoperative nausea and vomiting can necessitate readmission for hydration and symptomatic treatment.
- Infections: Wound infections or other postoperative infections may require hospital‐based management.
- Bile Leaks and Other Complications: Rare but serious complications such as bile leaks or intra‐abdominal collections can lead to readmission.

An Australian study by Pham, *et al*. demonstrated that unplanned readmissions could be reduced to as low as 2.5% with stringent patient selection and management protocols.^8^ In this study, significant pain was the primary reason for hospital return, emphasizing the importance of effective pain control strategies.^8^

By implementing comprehensive preoperative assessments and adhering to strict selection criteria, healthcare providers can minimize the risk of unplanned admissions and readmissions, ensuring that DCLC remains a safe and effective approach for appropriate patients.

#### Pain

Significant pain post‐surgery can often be a reason for delayed discharge and appropriate pain relief regimens form a key component in successful DCLC implementation.^36^

Choosing the optimal anaesthetic, analgesic, and perioperative anti‐emetic medications can decrease the intensity of postoperative pain, nausea, and vomiting, resulting in lower admission rates and improved outcomes for day‐only laparoscopic cholecystectomy patients.^27^

Kaushal‐Deep *et al*. compared the effectiveness of using 0.2% ropivacaine intra‐incisionally and intraperitoneally in patients undergoing uncomplicated laparoscopic cholecystectomy.^37^ They found that 31% of patients in the intraperitoneal group (*n* = 62) could be discharged as a day case compared to 48% in the intra‐incisional group (*n* = 68, *P* > 0.05).^37^ Combining the two techniques further increased the likelihood of same‐day discharge (*n* = 61, 89% of patients, *P* < 0.05) suggesting that the combined use of intra‐incisional and intraperitoneal ropivacaine is a cost‐effective way of improving DCLC.^37^

#### Patient quality of life and patient satisfaction

There have been mixed reports of patient satisfaction with some studies demonstrating no difference in DCLC and routine cholecystectomy^7^, ^13^, ^18^, ^23^, ^38^ and others demonstrating higher level of patient satisfaction in the day‐case group,^35^ specifically relating to; post‐operative nausea and vomiting score, median pain score, overall satisfaction, and time to return to work (96.6% DCLC *vs*. 86.2% overnight stay group).^37^

Four studies^13^, ^14^, ^18^, ^37^ focused on the number of patients satisfied with the surgery with no distinct comparison noting overall satisfaction rate in the day surgery group was significantly higher than that in the inpatient group, with a confidence interval of 95% (1.03–4.9).^23^

Assessing patient satisfaction remains difficult as there has been limited investigation using a standardized quality‐of‐life scoring tool. Some studies have used formal tools^20^, ^30^ where others have favoured a simple 1–10 subjective patient score.^10^

At present, there is need for further studies that stratify patient satisfaction by demographic in a standardized format which would aid in implementing patient selection criteria.

#### Barriers to implementation in Australia

Successful adoption of DCLC requires clear clinical pathways for clinical governance.

Proper patient selection is crucial in minimizing the chances of unplanned overnight admission or surgical review, achieving outcomes that are at least equivalent to current practices, and gaining acceptance from both surgeons and patients.^8^

In the Australian setting, there are challenges in implementing a standardized protocol for DCLC. Factors include mainly resource under‐availability with lack of dedicated day only wards, general understaffing, and no formal HITH staff or other trained staff to monitor patients after discharge.^39^ Blatt, *et al*. conducted their study in a regional teaching hospital which was discontinued due to the lack of necessary level of care for their patients.^39^

The rotational nature of medical training in public hospitals in Australia primarily affects registrars, not general surgeons, who are typically responsible for driving same‐day versus overnight stay practices. However, in rural settings, the presence of locum surgeons and a transient workforce can significantly impact staffing allocations and interpersonal relationships, leading to challenges in forming consistent and meaningful working partnerships with other clinicians.^39^ This variability can affect ‘buy‐in’ into departmental protocols and the adherence to available guidelines.^8^ The role of the surgeon extends beyond the operation itself, providing essential leadership in organizing and implementing these protocols, which includes staff and patient education. Strong individual preferences and biases of both surgeons and anaesthetists can also influence the consistency with which these protocols are followed.^8^

This was demonstrated Xiong *et al*. who showed that patient expectations of their post‐operative symptoms and care represented significant obstacle to the adoption of DCLC with some patients expressing anxiety of same‐day discharge or expecting overnight hospitalization to be a normal part of their recovery process.^20^

#### Cost

In the past 20 years, cholecystectomy has progressed from an open procedure requiring hospitalization for 4–5 days to a day case procedure for certain patients.^9^ DCLC offers patients the advantages of convenience and cost savings.^40^ For surgeons, performing DCLC in a dedicated day surgery unit can be more convenient as it allows for a more structured and predictable schedule. This setting often has fewer emergency interruptions compared to the main hospital operating rooms, where urgent and unplanned cases can disrupt scheduled procedures.^9^ This separation helps in maintaining a smoother workflow and better time management for elective surgeries. However, consideration must be given to the fact that not all hospital campuses have the facilities available for a dedicated daycare unit.^38^, ^41^

From as cost analysis standpoint, there have been many studies over the past two decades that have highlighted the specific financial savings that a conversion to a day case model can achieve. Ji *et al*. showed that the expenses incurred for surgery and hospitalization were 7235.7 RMB yuan (equivalent to 1575 AUD), representing a 17.5% reduction compared to the inpatient group.^42^ Victorzon, *et al*. reported that DCLC resulted in lower hospital costs ranging from 11% to 46% compared to inpatient treatment, with an average cost savings of 876 euros per patient (equivalent to 1422.92 Australian dollars).^43^ In 2006, Johansson *et al*. revealed that the average direct medical expenses per patient in the day‐care group were €3085, which was lower than the €3394 observed in the overnight group.^44^ This translates to a cost reduction of 10% per patient, which is equivalent to 501.85 Australian dollars. Even more significant cost savings associated with day‐case cholecystectomy have been shown. Similar findings were again shown by Topal *et al*. who observed that the mean cost per patient for in‐hospital treatment was 1309.35 €, whereas the cost for outpatient treatment was case 778.72 €. This represents a cost reduction of 40.5% in favour of day‐case cholecystectomy, which is equivalent to 860 Australian dollars.^45^

#### Recommendations for clinicians

**Fig. 2:**
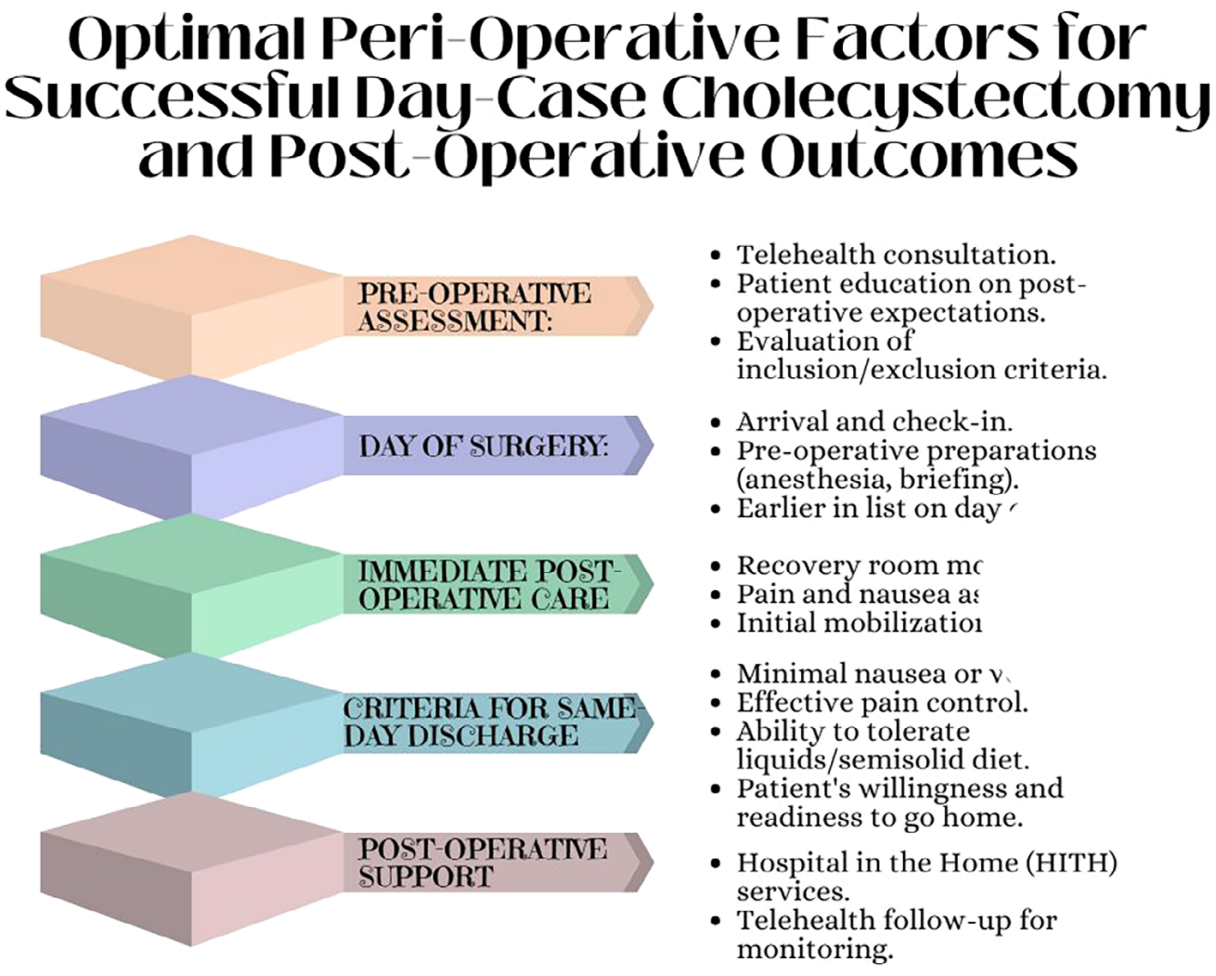
Summary of optimal peri‐operative factors for successful completion of DCLC.

The review has been distilled down to these best practices, based on the goal of providing the best patient outcomes and streamline the process of DCLC. These are summarized in Table 2 and Figure 2.

**Table 2:** Optimal peri‐operative factors for successful day‐case cholecystectomy and post‐operative outcomes

Factor	Description	References
Patient Education	Educating patients on post‐operative symptoms (pain, nausea, vomiting) to guide expectations	26
Analgesia Regimen	Use of combined intraincisional and intraperitoneal local anaesthetic, in addition to ongoing pain management post‐discharge.	36, 39
Surgeon Experience	Higher likelihood of same‐day discharge with specialist surgeons (HPB) and experienced consultants	25, 26, 29
Preoperative Telehealth	Reduces need for pre‐anaesthetic clinic visits	17
Scheduling	Early scheduling of procedures in the day	29
Post‐operative Evaluation	Evaluation of pain, nausea, ability to tolerate diet, and toileting post‐surgery	27, 30, 32
Post‐operative support	Use of Hospital in the Home (HITH) services and telehealth for follow‐up	16, 32, 33
Discharge Criteria	Minimal nausea, effective pain control, ability to tolerate diet, patient's willingness to go home	27, 30, 31, 32

This table outlines the peri‐operative factors that have been identified as optimal for ensuring the success of day‐case cholecystectomy procedures and favourable post‐operative outcomes. While these factors are considered best practices based on current evidence and clinical guidelines, they should not be interpreted as strict requirements. Additionally, it is important to recognize that this table may not encompass all studies relevant to each peri‐operative factor. The term ‘Clinical consensus’ indicates that the factor is widely accepted in the medical community through collective expert opinion rather than being tied to specific studies.

Summary of Best Practices

- **Preoperative**: Comprehensive patient selection and education are crucial.
- **Intraoperative**: Utilize experienced surgeons and optimal analgesia techniques.
- **Postoperative**: Ensure rigorous monitoring, effective pain management, and robust follow‐up support.

**Preoperative Phase:**

Patient Selection Criteria

- Elective Surgery: Ensure the procedure is elective and not an emergency.
- Age Considerations: Preferably select patients below 65 years of age.
- Health Status: Patients should have no significant underlying conditions (ASA grade I or II) and no prior upper abdominal surgeries.
- Proximity to Hospital: Patients should live within a reasonable distance from the hospital to facilitate quick return if necessary.
- Support System: Confirm the presence of a responsible adult to provide post‐discharge care.

Risk Assessment

- Common Bile Duct Stones: Utilize ultrasound and biochemical results to rule out high risk for common bile duct stones.
- Anaesthetic Risk: Evaluate patients for low anaesthetic risk (ASA grade I or II).

Preoperative Education

- Patient Education: Conduct thorough preoperative education sessions to set expectations regarding the procedure, recovery process, and discharge criteria.
- Informed Consent: Ensure informed consent is obtained after discussing potential risks and benefits.

**Intraoperative Phase:**

Surgical considerations

- Experienced Surgeons: Assign surgeries to specialist surgeons with extensive experience in laparoscopic cholecystectomy to improve outcomes.

Analgesia and Anaesthesia

- Optimal Analgesia: Use combined intra‐incisional and intraperitoneal local anaesthetic for effective pain management.
- Anaesthesia Management: Utilize anaesthetic techniques that facilitate rapid recovery and early mobilization.

**Postoperative Phase:**

Recovery Monitoring

- Immediate Postoperative Care: Monitor patients closely for pain, nausea, and other potential complications in recovery.
- Early Mobilization: Encourage early mobilization to promote faster recovery and reduce the risk of postoperative complications.

Discharge Criteria

- Pain Control: Ensure effective pain control is achieved using oral analgesics.
- Diet Tolerance: Verify that patients can tolerate oral intake without significant nausea or vomiting.
- Willingness to Go Home: Confirm that the patient feels comfortable and willing to be discharged.
- Criteria Check: Conduct a thorough check of all discharge criteria before releasing the patient.

Post‐Discharge Support

- Follow‐Up Care: Arrange for telehealth follow‐up consultations to monitor recovery and address any concerns.
- Hospital in the Home (HITH) Services: Provide access to HITH services for patients who may require additional support.

**Postoperative Outcomes and Follow‐Up:**

Monitoring and Management

- Symptom Tracking: Use telehealth services to track symptoms such as pain and nausea post‐discharge.
- Readmission Prevention: Establish protocols to quickly address any complications that may arise, reducing the risk of readmission.

Patient Satisfaction and Feedback

- Satisfaction Surveys: Collect patient feedback on their satisfaction with the DCLC process and outcomes.
- Continuous Improvement: Use feedback to continually refine and improve DCLC protocols.

By adhering to these recommendations, clinicians can enhance the safety and efficacy of DCLC, leading to better patient outcomes and increased satisfaction.

#### Further research implications

A shift to broadening potential inclusion criteria can be considered in future studies.^20^

To evaluate patient anxiety and satisfaction and to identify infrequent but significant differences between the two discharge practices, specially designed questionnaires such as the State–Trait Anxiety Inventory (STAI) Tool, or the Post‐operative Quality of Recovery Score (PQRS) could be utilized to attempt to form a standard tool for quantifying and comparing patient experiences.

Patient education is vital and should include guiding of post‐operative expectations.^29^

Future studies with high‐quality evidence such as randomized controlled trials will add further clarity to the benefits of DCLC and should include cost analysis to further quantify financial benefit.

## Conclusions

Conducting laparoscopic cholecystectomy as a day‐case procedure proves to be both safe and feasible, showcasing a satisfactory discharge rate and high levels of patient satisfaction without increasing postoperative complications. The critical role of patient selection is emphasized, especially in the preliminary stages of service development. As expertise advances, the selection criteria can be broadened to encompass a more diverse patient population.

The advantages of day‐case surgery, including shorter hospital stays and quicker returns to normal activities, justify patient preference and cost‐effectiveness. Institutions with dedicated day‐care capabilities, along with carefully selected patients, should consider implementing DCLC including some of the suggestions outlined in this article to enhance success.

Further randomized clinical trials are needed to diversify the body of knowledge in assessing the long‐term impact of DCLC on patients' quality of life, pain control, and return to normal activities and work.

## Author contributions

**Jamie Rickward:** Conceptualization; formal analysis; investigation; methodology; project administration; visualization; writing – original draft; writing – review and editing. **Iman Hameed:** Conceptualization; formal analysis; investigation; project administration; supervision; validation; writing – review and editing. **Simon Ho:** Conceptualization; formal analysis; methodology; supervision; writing – review and editing. **Shiran Wijeratne:** Supervision; writing – review and editing.

## Conflicts of interest

None declared.

## References

[1] Ahmad NZ , Byrnes G , Naqvi SA . A meta‐analysis of ambulatory versus inpatient laparoscopic cholecystectomy. Surg Endosc and other Interventional Tech. 2008; 22: 1928–1934. 10.1007/s00464-008-9867-2 18398648

[2] Australian Institute of Health and Welfare . Table 6.3: Interventions(a) reported for the 20 most common ACHI procedure blocks for overnight acute separations, public and private hospitals. Admitted patient care 2020–21: What procedures were performed? 6.3‐6.6. 2021.

[3] Australian Institute of Health and Welfare . Table 6.30: Time within which 50% of patients were admittted from public hospital elective surgery waiting lists, for the 25 most common intended procedures, by remoteness area of usual residence, 2020–21(a). Admitted patient care 2020–21: What procedures were performed? 6.3. 2021.

[4] Epari KP , Mukhtar AS , Fletcher DR , Samarasam I , Semmens JB . The outcome of patients on the cholecystectomy waiting list in Western Australia 1999–2005. ANZ J. Surg. 2010; 80: 673–764. 10.1111/j.1445-2197.2010.05428.x 21040330

[5] Australian Institute of Health and Welfare . Table 6.14: Patient days and average length of stay for emergency admissions involving surgery, public and private hospitals, 2021–22. Admitted patient care 2021–22 ‐ Australias Health What Procedures were performed? 2022.

[6] Vaughan J , Gurusamy KS , Davidson BR . Day‐surgery versus overnight stay surgery for laparoscopic cholecystectomy. Cochrane Database Syst. Rev. 2013; CD006798. 23904112 10.1002/14651858.CD006798.pub4 PMC11491877

[7] Gurusamy KS , Junnarkar S , Farouk M , Davidson BR . Day‐case versus overnight stay in laparoscopic cholecystectomy. Cochrane Database Syst. Rev. 2008; CD006798. 10.1002/14651858.CD006798.pub2 18254116

[8] Pham H , Chiong C , Sinclair JL *et al*. Day‐only elective cholecystectomy: early experience and barriers to implementation in Australia. ANZ J. Surg. 2021; 91: 590–596. 33369857 10.1111/ans.16526

[9] Voyles CR , Berch BR . Selection criteria for laparoscopic cholecystectomy in an ambulatory care setting. Surg. Endosc. 1997; 11: 1145–1146. 9373281 10.1007/s004649900556

[10] Tenconi SM , Boni L , Colombo EM , Dionigi G , Rovera F , Cassinotti E . Laparoscopic cholecystectomy as day‐surgery procedure: current indications and patients' selection. Int. J. Surg. 2008; 6: S86–S88. 19167938 10.1016/j.ijsu.2008.12.032

[11] Calland JF , Tanaka K , Foley E *et al*. Outpatient laparoscopic cholecystectomy: patient outcomes after implementation of a clinical pathway. Ann. Surg 2001; 233: 704–715. 11323509 10.1097/00000658-200105000-00015 PMC1421311

[12] Metcalfe MS , Mullin EJ , Maddern GJ . Relaxation of the criteria for day surgery laparoscopic cholecystectomy. ANZ J. Surg. 2006; 76: 142–144. 16626353 10.1111/j.1445-2197.2006.03672.x

[13] Hollington P , Toogood GJ , Padbury RTA . A prospective randomized trial of day‐stay only versus overnight‐stay laparoscopic cholecystectomy. Aust. N. Z. J. Surg. 1999; 69: 841–843. 10613279 10.1046/j.1440-1622.1999.01713.x

[14] Keulemans Y , Eshuis J , De Haes H , De Wit LT , Gouma DJ . Laparoscopic cholecystectomy: day‐care versus clinical observation. Ann. Surg. 1998; 228: 734–740. 9860471 10.1097/00000658-199812000-00003 PMC1191590

[15] Kumar S , Ali S , Ahmad S , Meena K , Chandola HC . Randomised controlled trial of day‐case laparoscopic cholecystectomy vs routine laparoscopic cholecystectomy. Indian J. Surg. 2015; 77: 520–524. 26730057 10.1007/s12262-013-0906-4 PMC4692952

[16] Mjåland O , Reder J , Aasboe V , Trondsen E , Buanes T . Outpatient laparoscopic cholecystectomy. Br. J. Surg. 2005; 84: 958–961. 10.1002/bjs.1800840714 9240135

[17] Urbonas T , Lakha AS , King E *et al*. The safety of telemedicine clinics as an alternative to in‐person preoperative assessment for elective laparoscopic cholecystectomy in patients with benign gallbladder disease: a retrospective cohort study. Patient Saf. Surg. 2023; 17: 23. 37644474 10.1186/s13037-023-00368-7 PMC10466851

[18] Kaman L . Day care laparoscopic cholecystectomy: next standard of Care for Gallstone Disease. Gastroenterology Res. 2011; 4: 257–261. 27957025 10.4021/gr374w PMC5139863

[19] El‐Sharkawy AM , Tewari N , Vohra RS *et al*. The cholecystectomy As a day case (CAAD) score: a validated score of preoperative predictors of successful day‐case cholecystectomy using the CholeS data set. World J. Surg. 2019; 43(8): 1871–2115. 31016355 10.1007/s00268-019-04981-5 PMC9883331

[20] Xiong W , Li M , Wang M , Zhang S , Yang Q . The safety of laparoscopic cholecystectomy in the day surgery unit comparing with that in the inpatient unit: a systematic review and meta‐analysis. Biomed. Res. Int. 2020; 2020: 1924134. 32420324 10.1155/2020/1924134 PMC7206864

[21] Zaafouri H , Mrad S , Khedhiri N , Haddad D , Bouhafa A , Maamer AB . First experience with outpatient laparoscopic cholecystectomy in Tunisia. Pan. Afr. Med. J. 2017; 28: 78. 29255548 10.11604/pamj.2017.28.78.9564 PMC5724953

[22] Cullinane C , Fullard A , Croghan SM , Elliott JA , Fleming CA . Effect of obesity on perioperative outcomes following gastrointestinal surgery: meta‐analysis. BJS Open. 2023; 7(4): zrad026. 37428558 10.1093/bjsopen/zrad026 PMC10332403

[23] Tjeertes EEKM , Hoeks SSE , Beks SSBJC , Valentijn TTM , Hoofwijk AAGM , Stolker RJRJ . Obesity – a risk factor for postoperative complications in general surgery? BMC Anesthesiol. 2015; 15: 112. 26228844 10.1186/s12871-015-0096-7 PMC4520073

[24] Robinson TN , Biffl WL , Moore EE *et al*. Predicting failure of outpatient laparoscopic cholecystectomy. Am. J. Surg. 2002; 184: 515–518. 12488152 10.1016/s0002-9610(02)01080-2

[25] Tandon A , Sunderland G , Nunes Q , Misra N , Shrotri M . Day case laparoscopic cholecystectomy in patients with high BMI: experience from a UK centre. Ann. R. Coll. Surg. Engl. 2016; 98: 329–333. 27087326 10.1308/rcsann.2016.0125 PMC5227041

[26] Ammori BJ , Larvin M , McMahon MJ . Elective laparoscopic cholecystectomy: preoperative prediction of duration of surgery. Surg. Endosc. 2001; 15: 297–300. 11344433 10.1007/s004640000247

[27] Al‐Qahtani HH , Alam MK , Asalamah S , Akeely M , Ibrar M . Day‐case laparoscopic cholecystectomy. Saudi Med. J. 2015; 36: 46–51. 25630004 10.15537/smj.2015.1.9738 PMC4362199

[28] Psaila J , Agrawal S , Fountain U *et al*. Day‐surgery laparoscopic cholecystectomy: factors influencing same‐day discharge. World J. Surg. 2008; 32: 76–81. 17990027 10.1007/s00268-007-9225-x

[29] Vuilleumier H , Halkic N . Laparoscopic cholecystectomy as a day surgery procedure: implementation and audit of 136 consecutive cases in a university hospital. World J. Surg. 2004; 28: 737–740. 15457349 10.1007/s00268-004-7376-6

[30] Campbell M , Ng D , Albatat B , Lowen D , Bird D , Hodgson R . Quality of recovery assessment of day case and multiday stay patients undergoing elective laparoscopic cholecystectomy. Turk J Surg. 2021; 37: 355–362. 35677494 10.47717/turkjsurg.2021.5451 PMC9130945

[31] Barthelsson C , Anderberg B , Ramel S , Björvell C , Giesecke K , Nordström G . Outpatient versus inpatient laparoscopic cholecystectomy: a prospective randomized study of symptom occurrence, symptom distress and general state of health during the first post‐operative week. J. Eval. Clin. Pract. 2008; 14: 577–584. 18462280 10.1111/j.1365-2753.2007.00920.x

[32] Akoh JA , Watson WA , Bourne TP . Day case laparoscopic cholecystectomy: reducing the admission rate. Int. J. Surg. 2011; 9: 63–67. 20887821 10.1016/j.ijsu.2010.09.002

[33] Briggs C , Irving G , Mann C *et al*. Introduction of a day‐case laparoscopic cholecystectomy service in the UK: a critical analysis of factors influencing same‐day discharge and contact with primary care providers. Ann. R. Coll. Surg. Engl 2009; 91: 583–590. 19558787 10.1308/003588409X432365 PMC2966163

[34] Sandblom G , Videhult P , Crona Guterstam Y , Svenner A , Sadr‐Azodi O . Mortality after a cholecystectomy: a population‐based study. HPB 2015; 17: 239–243. 25363135 10.1111/hpb.12356 PMC4333785

[35] Salleh AAM , bin Ariffin AC , Hairol O *et al*. Randomized controlled trial comparing daycare and overnight stay laparoscopic cholecystectomy. Clin. Ter. 2015; 166: e165–e168. 26152626 10.7417/CT.2015.1848

[36] Curet MJ , Contreras M , Weber DM , Albrecht R . Laparoscopic cholecystectomy: outpatient vs inpatient management. Surgical Endosc and other Interventional Tech. 2002; 16: 453–457. 10.1007/s00464-001-8129-3 11928027

[37] Kaushal‐Deep SM , Lodhi M , Anees A , Khan S , Khan MA . Randomised prospective study of using intraoperative, intraincisional and intraperitoneal ropivacaine for the early discharge of post‐laparoscopic cholecystectomy patients as a day case in a cost‐effective way in government setup of low‐income and middle‐income countries: opening new horizons. Postgrad. Med. J. 2019; 95: 78–84. 31015318 10.1136/postgradmedj-2018-135662

[38] Bal S , Reddy LGS , Parshad R , Guleria R , Kashyap L . Feasibility and safety of day care laparoscopic cholecystectomy in a developing country. Postgrad. Med. J. 2003; 79: 284–288. 12782776 10.1136/pmj.79.931.284 PMC1742692

[39] Blatt A , Chen S . Day‐only laparoscopic cholecystectomy in a regional teaching hospital. ANZ J. Surg. 2003; 73: 321–325. 12752289 10.1046/j.1445-2197.2003.t01-1-02614.x

[40] Skattum J , Edwin B , Trondsen E , Mjåland O , Raeder J , Buanes T . Outpatient laparoscopic surgery: feasibility and consequences for education and health care costs. Surgical endoscopy and other interventional. Dent. Tech. 2004; 18: 796–801. 10.1007/s00464-003-9180-z 15216863

[41] Chauhan A , Mehrotra M , Bhatia PK , Baj B , Gupta AK . Day care laparoscopic cholecystectomy: a feasibility study in a public health service hospital in a developing country. World J. Surg. 2006; 30: 1690–1965. 16902738 10.1007/s00268-006-0023-7

[42] Ji W , Ding K , Li LT , Wang D , Li N , Li JS . Outpatient versus inpatient laparoscopic cholecystectomy: a single center clinical analysis. Hepatobiliary Pancreat. Dis. Int. 2010; 9: 60–64. 20133231

[43] Victorzon M , Tolonen P , Vuorialho T . Day‐case laparoscopic cholecystectomy: treatment of choice for selected patients? Surgical endoscopy and other interventional. Dent. Tech. 2007; 21: 70–73. 10.1007/s00464-005-0787-0 17001441

[44] Johansson M , Thune A , Nelvin L , Lundell L . Randomized clinical trial of day‐care versus overnight‐stay laparoscopic cholecystectomy. Br. J. Surg. 2006; 93: 40–45. 16329083 10.1002/bjs.5241

[45] Topal B , Peeters G , Verbert A , Penninckx F . Outpatient laparoscopic cholecystectomy: clinical pathway implementation is efficient and cost effective and increases hospital bed capacity. Surg. Endosc. 2007; 21: 1142–1146. 17237916 10.1007/s00464-006-9083-x

